# A case of endoscopic full‐thickness resection for gastric gastrointestinal stromal tumor in the submucosal tunnel

**DOI:** 10.1002/deo2.282

**Published:** 2023-08-27

**Authors:** Hironari Shiwaku, Hiroki Okada, Akio Shiwaku, Hiroshi Kusaba, Kenji Maki, Hideki Shimaoka, Yasuhiro Hashimoto, Teppei Yamada, Fumihiro Yoshimura, Suguru Hasegawa

**Affiliations:** ^1^ Department of Gastroenterological Surgery Fukuoka University Faculty of Medicine Fukuoka Japan

**Keywords:** endoscopic full‐thickness resection, endoscopic suturing, gastrointestinal stromal tumor, pocket‐creation method, third space endoscopy

## Abstract

The patient was a 49‐year‐old female with a submucosal tumor (12×12 mm) located in the lesser curvature side of the stomach. The diagnosis by endoscopic ultrasound fine‐needle aspiration was of a gastrointestinal stromal tumor. Computed tomography and endoscopic ultrasound showed gastrointestinal stromal tumor with an intra‐luminal growth type. Endoscopic full‐thickness resection was then performed. To achieve good counter traction, enough safety margin, and minimal defect of muscle, full‐thickness resection via creating a submucosal tunnel was performed as a new technique. The final histological diagnosis was gastrointestinal stromal tumor with R0 resection.

## INTRODUCTION

Recently, endoscopic full‐thickness resection (EFTR) has been performed for gastrointestinal stromal tumors (GISTs) but there have been no reports of EFTR using a submucosal tunnel‐like third space endoscopy. In this report, we present a case of EFTR for gastric GIST using a method of creating a submucosal tunnel (Video S1).

## CASE REPORT

**FIGURE 1:**
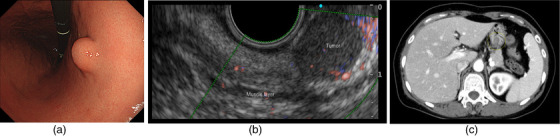
(a) The submucosal tumor (12×12 mm) located in the lesser curvature side of the stomach. (b) Endoscopic ultrasound showed an irregular low‐echoic lesion connected to the muscle layer. (c) Computed tomography showed a low‐density mass in the stomach. The tumor size was 12 × 12 mm. There was no tumor metastasis.

**FIGURE 2:**
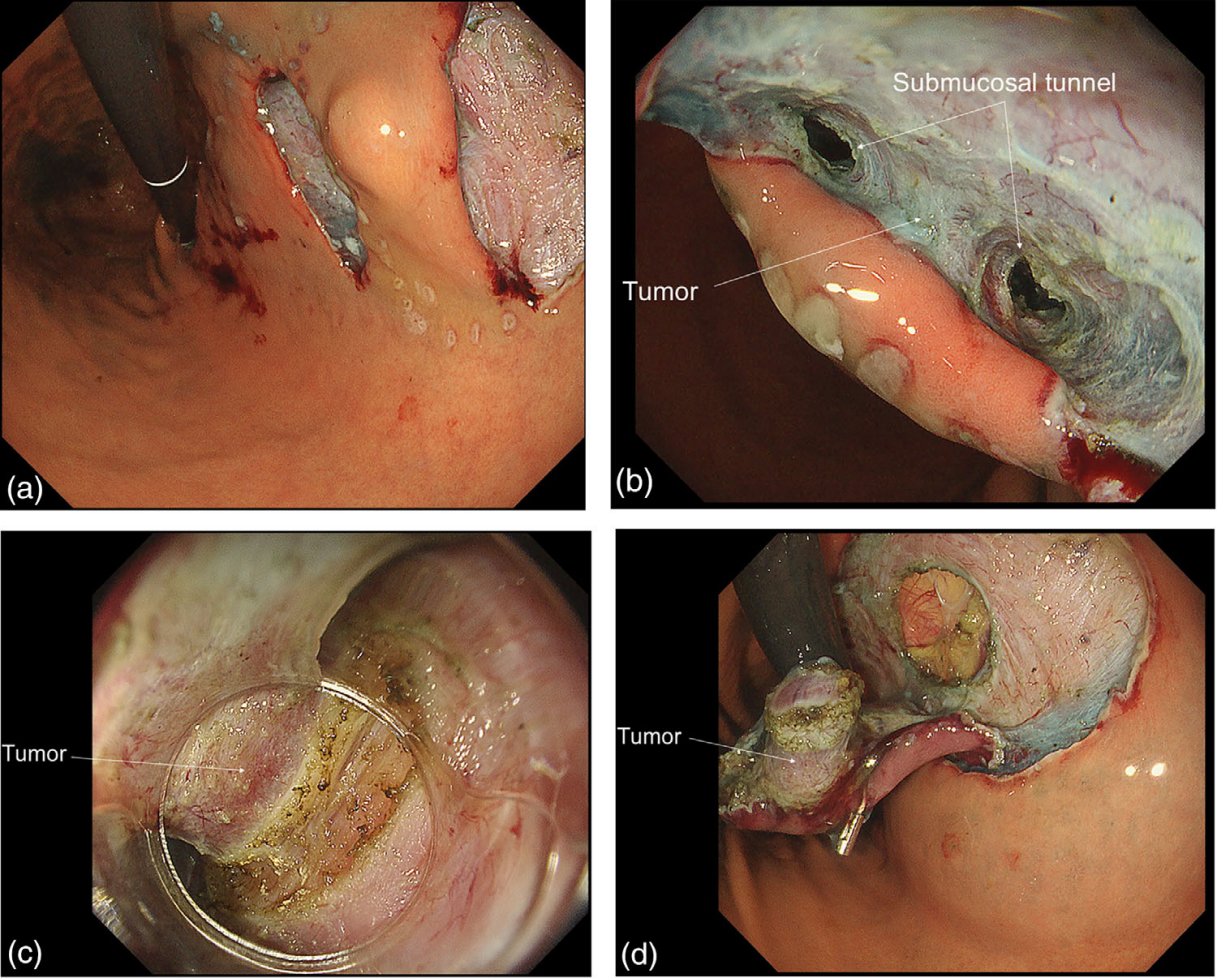
(a) Mucosal incisions were made on the oral and anal sides of the tumor. (b) An endoscopic image from the oral side of the tumor. The submucosal tunnels were roughly created on the anterior and posterior sides of the tumor. (c) Circumferential incisions were made to the muscle layer after keeping a distance from the tumor. (d) Endoscopic image taken immediately after full‐thickness resection. The tumor was resected en bloc.

**FIGURE 3:**
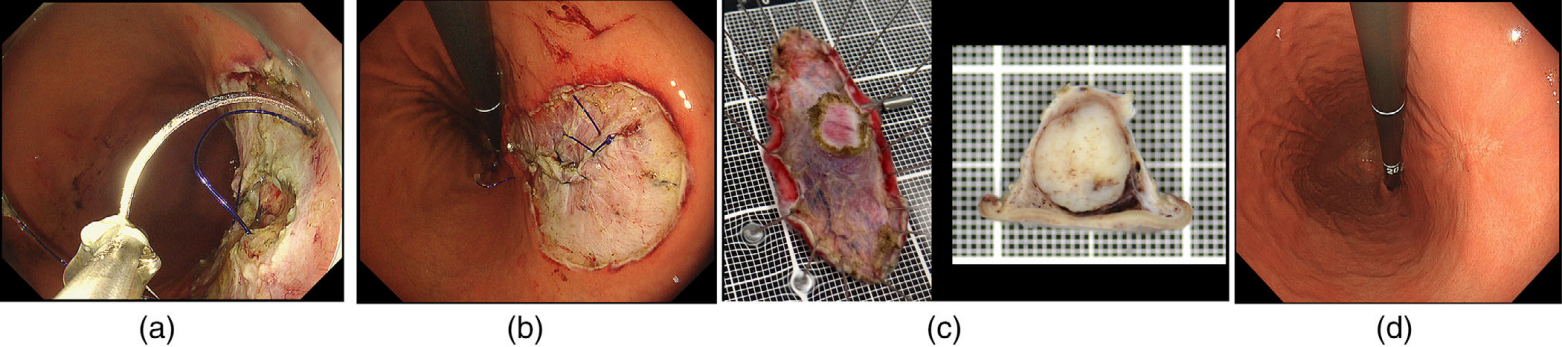
(a) The muscle defect was sutured using an endoscopic suturing device. (b) The muscle defect was completely closed. (c) The resected specimen from endoscopic full‐thickness resection. The tumor was completely resected without exposure. The final histological diagnosis was a very low‐grade gastrointestinal stromal tumor (GIST) in accordance with the Modified Fletcher classification. (d) After 1 year of treatment, there has been no recurrence observed.

The patient was a 49‐year‐old female with a submucosal tumor in the stomach that was detected by screening esophagogastroduodenoscopy. The submucosal tumor (12 × 12 mm) was located in the lesser curvature side of the stomach. (Figure 1a). Histological diagnosis by endoscopic ultrasound fine‐needle aspiration was GIST. Computed tomography and endoscopic ultrasound showed GIST with an intra‐luminal growth type (Figure 1b,c). Next, EFTR was performed on this patient (Figures 2 and 3). Written informed consent was obtained from the patient. This study was conducted according to the principles of the Declaration of Helsinki.

As there was a possibility of a shift to emergency surgery, we inserted a few ports into the abdomen and placed a clip to clamp the upper jejunum and prevent CO_2_ from filling the gastrointestinal tract before performing EFTR.

After marking the area around the submucosal tumor, saline was injected locally, and mucosal incisions were made on the oral and anal sides of the tumor. Submucosal tunnels were then roughly created on the anterior and posterior sides of the tumor. The area of the continuous part of the tumor and the muscle layer was narrowed down as much as possible so that the muscle layer defect was as small as possible (the part to be sutured was minimized). These procedures were performed in the submucosal tunnel and were performed smoothly and safely with stable visibility and good countertraction. An EndoTrac (Top Corporation) was attached to the lesion to ensure the continuation of the procedure even in the event of gastric collapse caused by full‐thickness resection. After keeping a safe distance from the tumor and incising the muscle layer circumferentially, the tumor was resected en bloc and safely removed with a net. After closing the defect in the muscle tightly by continuous suture using an endoscopic needle holder (FG‐260, SutuArt; Olympus Medical Systems), we finished the endoscopic procedure.

Because the tumor was located on the lesser curvature of the stomach, it could not be confirmed from the laparoscopic side; however, a small amount of emphysema was observed on the lesser curvature side of the stomach after EFTR.

After removing the clip that clamped the upper jejunum and port, the procedure was finished without any complications. The patient was discharged within a few days without major complications. The final histological diagnosis was very low‐grade GIST in accordance with the Modified Fletcher classification, and margins were tumor‐free (Figure 3c). One year has passed since the treatment, and no recurrence has been observed (Figure 3d).

## DISCUSSION

**FIGURE 4:**
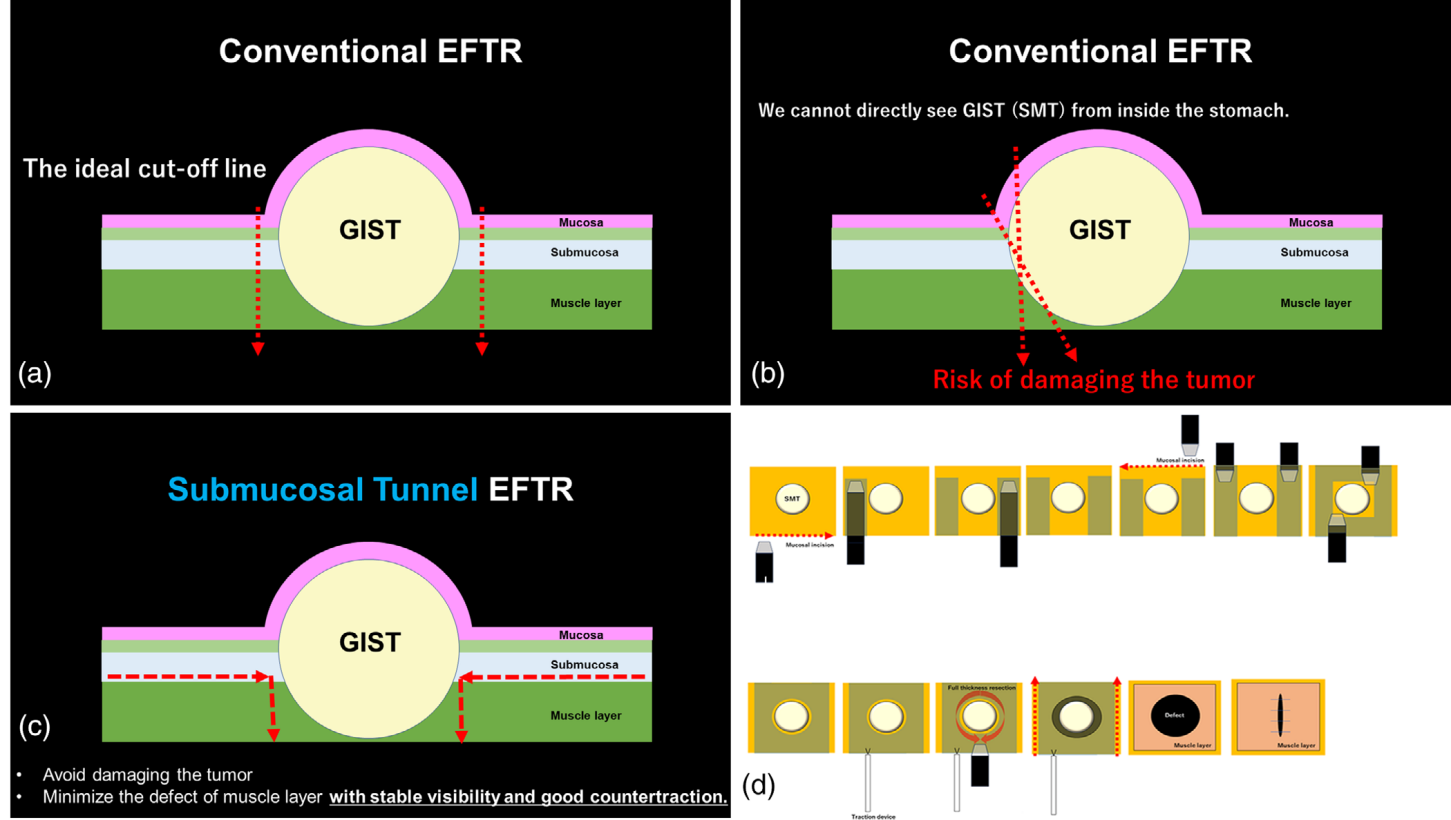
(a) The ideal cut‐off line of conventional endoscopic full‐thickness resection (EFTR). (b) One reason for postoperative recurrence is that submucosal tumors cannot be directly visualized from inside the lumen, so it has been thought that incisions near the tumor were made when EFTR was performed. (c) If the submucosal tunneling technique is used in EFTR, we can avoid damaging the tumor and minimize the defect of the muscle layer with stable visibility and good countertraction. (d) The Image of EFTR using a submucosal tunnel. Mucosal incisions are made on the oral and anal sides of the tumor. Submucosal tunnels are roughly created on the anterior and posterior sides of the tumor. The creation of the submucosal tunnel may be accomplished from one side. The area of the continuous part of the tumor and the muscle layer is narrowed down as much as possible so that the muscle layer defect is as small as possible (the part to be sutured was minimized). The traction device is attached to the lesion to ensure the continuation of the procedure even in the event of gastric collapse caused by full‐thickness resection. After keeping a safe distance from the tumor and incising the muscle layer circumferentially, the tumor was resected en bloc and safely. After performing mucosal incisions on the anterior and posterior walls, the tumor, covered by mucosa and surrounding tissues, is extracted outside the body. The defect in the muscular layer is tightly closed using endoscopic closure devices such as endoscopic needle holders or clips. If possible, mucosal closure is also performed, but it is not mandatory.

Recently, EFTR has been performed for small‐sized GISTs.^1^, ^2^ However, it has been pointed out that EFTR by the conventional method tends to cause positive tumor margin by pathological evaluation and has problems of postoperative recurrence.^3^, ^4^. One reason is that submucosal tumors cannot be directly visualized from inside the lumen, so it has been thought that incisions near the tumor were made when EFTR was performed (Figure 4).

Therefore, we created a submucosal tunnel around the tumor from the oral and anal sides and then performed EFTR in a manner that did not damage the tumor while visually observing the tumor in the submucosal tunnel (Figure 4).

Creating a submucosal tunnel and performing endoscopic treatment are methods used in so‐called third space endoscopy, such as peroral endoscopic myotomy, peroral endoscopic tumor resection (POET), submucosal tunneling endoscopic resection or endoscopic submucosal dissection by the pocket creation method.^5^, ^6^, ^7^, ^8^, ^9^

Advantages of using a submucosal tunnel include 1) sufficient countertraction during the procedure, 2) the ability to directly visualize the tumor, 3) precise layer detachment that does not expose the tumor, and 4) precise manipulation to minimize defects in the muscle layer (Video S1). Additionally, the operation can proceed in the submucosal tunnel with a small amount of air supply from the endoscope. Therefore, even if the lumen collapses during full‐thickness resection, the operation can proceed in the submucosal tunnel. Furthermore, when a traction device is attached to the lesion, a more stable vision is ensured.

There may be opinions suggesting that POET (or submucosal tunneling endoscopic resection) could be a viable treatment option for this case. Indeed, one of the advantages of POET is the ease of wound closure and the absence of mucosal defects, which eliminates concerns about postoperative stenosis. However, since POET is the enucleation of the tumor, there may be a risk of postoperative recurrence due to damaging the tumor, especially in the case of GISTs. In this particular case of gastric GIST, there was no concern about postoperative stenosis. Considering that GISTs are fragile tumors with pseudocapsule, and the expandability of the gastric mucosa is limited compared to the esophagus, performing POET could raise concerns about the risk of damaging the tumor during the dissection between the tumor and mucosa. Therefore, in this case, the decision was made to choose EFTR using a method of creating a submucosal tunnel according to the histological diagnosis of endoscopic ultrasound fine‐needle aspiration. Consequently, it was possible to safely remove the tumor without causing any damage to it.

As for indications of this method, intra‐luminal growth types of GISTs smaller than 30 mm are considered good candidates.^1^ Regarding the tumor's location, similar to the pocket creation method in ESD,^7^, ^10^ this technique can be applied wherever submucosal tunnels can be created.

In summary, we reported the first clinical case of using a novel EFTR technique that involved creating a submucosal tunnel for gastric GIST. Although it has the drawback of causing larger mucosal defects than conventional EFTR, it is a good method with excellent safety and stability in endoscopic manipulation (Video S1). Overall, this method is particularly useful in regions where submucosal tunnels can be created.

## CONFLICT OF INTEREST STATEMENT

None.

## Supporting information

The procedure of this report is summarized in the supporting video.
